# Human bronchial fibroblasts express the 5-lipoxygenase pathway

**DOI:** 10.1186/1465-9921-7-102

**Published:** 2006-07-27

**Authors:** Anna J James, John F Penrose, Angelica M Cazaly, Stephen T Holgate, Anthony P Sampson

**Affiliations:** 1 Division of Infection, Inflammation and Repair (IIR), University of Southampton, School of Medicine, Southampton General Hospital, Southampton, SO16 6YD, UK

## Abstract

**Background:**

Fibroblasts are implicated in sub-epithelial fibrosis in remodeled asthmatic airways and contribute to airway inflammation by releasing cytokines and other mediators. Fibroblast activity is influenced by members of the leukotriene family of bronchoconstrictor and inflammatory mediators, but it is not known whether human bronchial fibroblasts can synthesize leukotrienes.

**Methods:**

The expression of leukotriene biosynthetic enzymes and receptors was investigated in primary fibroblasts from the bronchi of normal and asthmatic adult subjects using RT-PCR, Western blotting, immunocytochemistry and flow cytometry.

**Results:**

These techniques revealed that human bronchial fibroblasts from both subject groups constitutively express 5-lipoxygenase, its activating protein FLAP, the terminal enzymes leukotriene A_4 _hydrolase and leukotriene C_4 _synthase, and receptors for leukotriene B_4 _(BLT1) and cysteinyl-leukotrienes (CysLT_1_). Human bronchial fibroblasts generated immunoreactive leukotriene B_4 _and cysteinyl-leukotrienes spontaneously and in increased amounts after calcium-dependent activation. Flow cytometry showed that human bronchial fibroblasts transformed to a myofibroblast-like phenotype by culture with transforming growth factor-β_1 _expressed 320–400% more immunofluorescence for leukotriene C_4 _synthase and CysLT_1 _receptors, with 60–80% reductions in leukotriene A_4 _hydrolase and BLT1 receptors.

**Conclusion:**

These results indicate that human bronchial fibroblasts may not only respond to exogenous leukotrienes but also generate leukotrienes implicated in narrowing, inflammation and remodeling of the asthmatic airway.

## Background

Airway inflammation and remodeling are prominent features of the asthmatic lung, characterized by leukocyte infiltration, oedema and mucus hypersecretion, combined with sub-epithelial fibrosis and smooth muscle hypertrophy. Fibroblasts are the most abundant cell type of the lung interstitium and play a significant role in airway inflammation by expressing an array of cytokines and adhesion molecules which play key roles in infiltration and activation of eosinophils and other leukocytes [1]. Fibroblasts can be transformed into the myofibroblast phenotype, which express contractile proteins and have an increased capacity for collagen deposition, in a process regulated by cytokines, growth factors and matrix components [2]. Myofibroblasts are also critical to the development of sub-epithelial fibrosis, which is due to enhanced accumulation of the repair-like proteins fibronectin, tenascin, and collagens (types I, III and V) [3]. Increased numbers of myofibroblasts are found beneath the bronchial epithelium of asthmatic patients compared to normal subjects, and their numbers correlate with the degree of collagen deposition [4].

Leukotrienes are a family of lipid inflammatory mediators of central importance to the pathogenesis of asthma. As well as inducing bronchoconstriction, airway oedema, mucus secretion and eosinophil infiltration [5], they may also play a role in remodeling. Members of the cysteinyl-leukotriene sub-family increase the proliferation of bronchial epithelial cells and airway smooth muscle cells [6,7]. Leukotrienes also increase the proliferation of fibroblasts and enhance their release of collagens and collagenase [8,9]. In a murine model of chronic allergic asthma with fibrosis, blockade of cysteinyl-leukotriene activity by montelukast significantly reduced eosinophil infiltration, mucus plugging, and smooth muscle hyperplasia, and completely prevented the development of sub-epithelial fibrosis [10]. Mice depleted of leukotrienes by deletion of the 5-lipoxygenase (5-LO) gene are protected against lung fibrosis induced by bleomycin [11]. In asthmatic patients, bronchoconstrictor responses to allergen and other asthma triggers are associated with rapid *de novo *synthesis of leukotrienes by airway mast cells, eosinophils and other leukocytes, but the cellular sources of leukotrienes involved in airway remodeling are not clear. We postulate that human bronchial fibroblasts (HBFs) themselves are capable of generating leukotrienes that may influence fibrosis and other remodeling processes by autocrine or paracrine mechanisms.

Leukotriene synthesis is most apparent in cells of myeloid origin, in which activation and calcium influx leads to the liberation of arachidonic acid from cell membranes by phospholipase A_2_. Arachidonate is donated to 5-LO by the 5-LO activating protein (FLAP) and oxidized to the unstable epoxide, leukotriene (LT)A_4_. In some cell types, the predominant activity of LTA_4 _hydrolase generates LTB_4_, while in other cell-types the integral membrane protein LTC_4 _synthase conjugates LTA_4 _with glutathione to provide the first of the cysteinyl-leukotrienes, LTC_4_. After carrier-mediated export, LTC_4 _with its extracellular metabolites LTD_4 _and LTE_4 _exert bronchoconstrictor and pro-inflammatory activity at specific G-protein coupled receptors termed cysteinyl-leukotriene receptor type 1 (CysLT_1_) [12]. LTB_4 _acts chemotactically at high-affinity BLT1 receptors [13].

We therefore investigated whether HBFs from normal and asthmatic subjects can express mRNA and protein for the 5-LO pathway enzymes and generate biologically-active leukotrienes, either constitutively or after treatment with autacoids, cytokines and growth factors.

## Methods

### Reagents

Materials were purchased as follows: Biotinylated swine anti-rabbit antibody, biotinylated rabbit anti-mouse antibody, streptavidin-biotin-horseradish-peroxidase conjugate, rabbit and goat sera, rabbit immunoglobulins and mouse IgG_1 _were from DAKO Ltd (High Wycombe, Bucks, UK). Omniscript RT-PCR kits were purchased from Qiagen (Crawley, UK), goat anti-rabbit/PE, rabbit anti-goat/FITC and rabbit anti-mouse/FITC antibodies from Southern Biotechnologies Ltd (Birmingham, Alabama, USA), and Biotrak EIA kits for LTC_4_/D_4_/E_4 _and for LTB_4 _from Amersham Pharmacia Biotech (Little Chalfont, Bucks, UK). DMEM, penicillin/streptomycin, Trizol, FCS, EGF, human serum, and trypsin/EDTA were from Gibco Life Technologies Ltd (Paisley, UK). TGF-β_1_, protease inhibitor cocktail, calcium ionophore A23187, saponin, dexamethasone, histamine, bradykinin and antibodies against actin, myosin and vimentin were from Sigma Chemical Co. (Poole, Dorset, UK). Interleukin (IL)-1β, tumor necrosis factor-α (TNF-α) and IFN-γ were from R&D Systems (Abingdon, UK). Bis-acrylamide solution, sodium dodecyl sulphate and molecular weight markers were from Bio-Rad (Hemel Hempstead, UK). Polyclonal antibodies recognizing the C-terminal amino acids 331–352 of the BLT1 receptor and the C-terminal amino acids 318–337 of the CysLT_1 _receptor were purchased from Cayman Chemical (Ann Arbor, MI, USA), as were the CysLT1 receptor blocking peptide, LTB_4_, LTD_4 _and MK-571. Rabbit antisera directed against 5-LO, FLAP, LTA_4 _hydrolase [14], goat antibody to the whole human CysLT_1_R protein [15] and human 5-LO and FLAP protein standards were kind gifts from Dr JF Evans (Merck & Co., West Point, Pa, USA). LTC_4 _synthase antibody does not cross-react with FLAP or with microsomal glutathione-S-transferase type II (MGST-II) [16].

### Culture of primary human lung fibroblasts

Fibroblasts were grown as described [17] from explants of bronchial biopsy tissue from normal, non-atopic, non-smoking adult volunteers (4 males, 1 female) with a mean forced expiratory volume in one second (FEV_1_) of 95.8 ± 2.1% of predicted and a provocation concentration of inhaled histamine to reduce FEV_1 _by 20% (histamine PC_20_) >32 mg/ml, and from mild-moderate adult asthmatic donors (3 male, 2 female) with mean FEV_1 _of 76.4 ± 2.7% of predicted and geometric mean histamine PC_20 _of 2.24 mg/ml (95% confidence interval 0.71 – 7.94). Subjects had been free of respiratory tract infections for at least 4 weeks before the study. All subjects gave written informed consent to the study, which was in compliance with the Helsinki Declaration and approved by the Southampton & Southwest Hampshire Local Research Ethics Committee (130/98). Six submucosal biopsies from each subject were obtained as described [17]; biopsies were cut into pieces with sterile scalpel blades in a Petri dish containing DMEM (containing 10% FCS, 50 U/ml penicillin, 50 μg/ml streptomycin and 2 mM L-glutamine). The tissues were cultured in a humidified incubator (37°C, 5% CO_2_) for approximately one week, during which fibroblasts migrated from the tissue and proliferated on the base of the culture dish. Separate cultures were maintained from each donor; cultures were fed every 2–3 days and passaged weekly. Cells for experiments were between passages five and seven for both normal and asthmatic donors. Before experimentation, cells were incubated in Ultraculture serum-free medium (BioWhittaker, Wokingham, UK) for 24 hours. The cells were characterized by flow cytometry using antibodies against the mesenchymal cell marker vimentin and the myofibroblast cell marker α-smooth muscle actin. All cell lines were uniformly immunopositive for vimentin (Figure 2) and had consistently low levels of α-smooth muscle actin (Figure 4), indicating that the cultures were uncontaminated fibroblasts.

**Figure 1:**
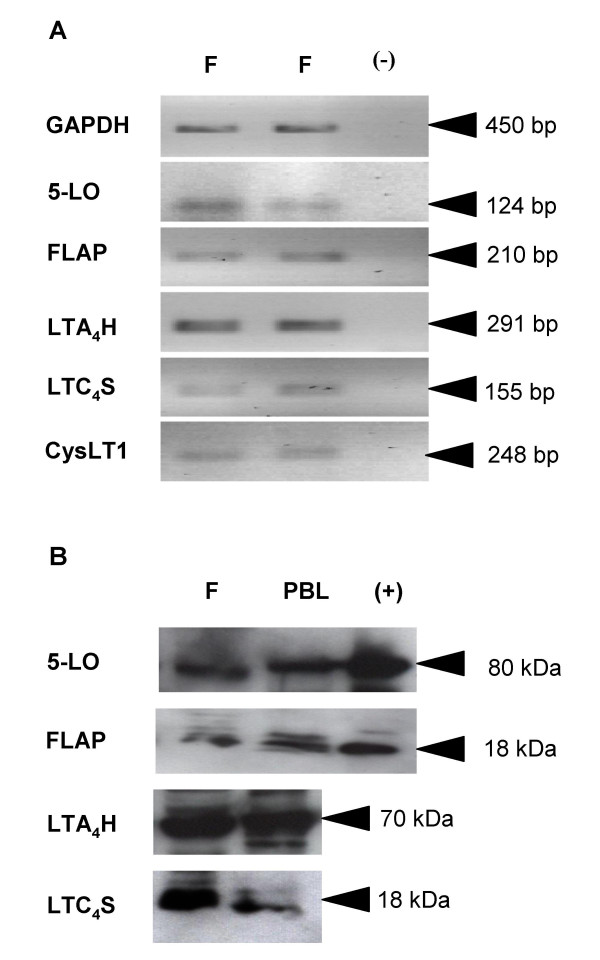
**Constitutive expression of LT pathway enzyme mRNA and protein in human bronchial fibroblasts**. (A) RT-PCR analyses of human bronchial fibroblasts (F) from a representative normal donor in duplicate to show equal loading. GAPDH was the internal positive control, and PCR was carried out in the absence of RT product as a negative control (-). The amplicons generated were consistent with the sizes expected for GAPDH (450 bp), 5-LO (124 bp), FLAP (210 bp), LTA_4_H (291 bp), LTC_4_S (155 bp) and CysLT_1 _(248 bp). Panel (B) shows an immunoblot of lysates from fibroblasts from a representative asthmatic patient (F) compared with peripheral blood leukocytes (PBL). Each lane represents the same amount of total cellular protein. Recombinant protein standards are shown for 5-LO and FLAP (+). n = 5 normals, n = 5 asthmatics.

**Figure 2:**
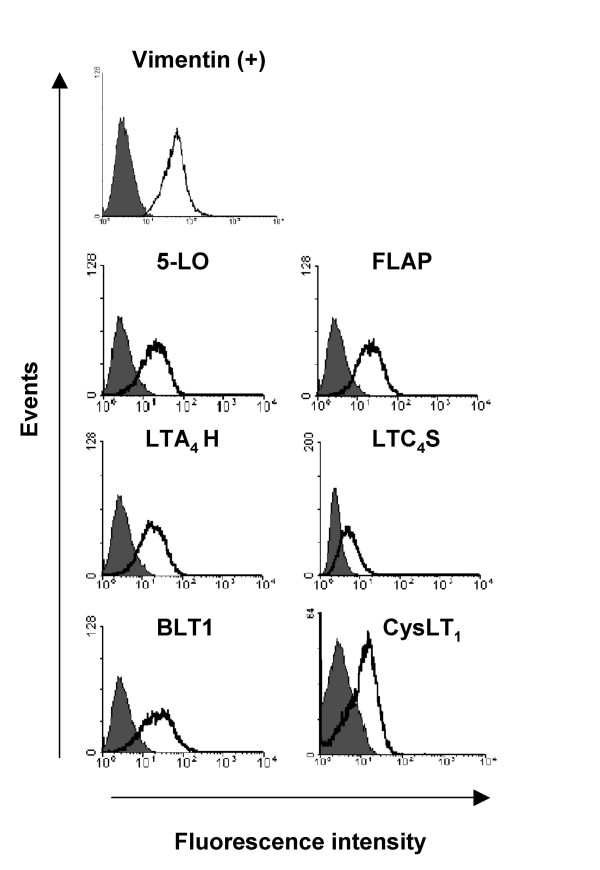
**Flow cytometry of baseline expression of 5-LO pathway enzymes and LT receptors in human bronchial fibroblasts**. Flow cytometric histograms showing specific immunostaining for the cytoskeletal protein vimentin as a positive control, 5-LO, FLAP, LTA_4_H, LTC_4_S, BLT1 and CysLT_1_R (open curves) in fibroblasts from one representative normal donor compared to the relevant isotype controls (filled curves). n = 5 normals, n = 4 asthmatics.

**Figure 3:**
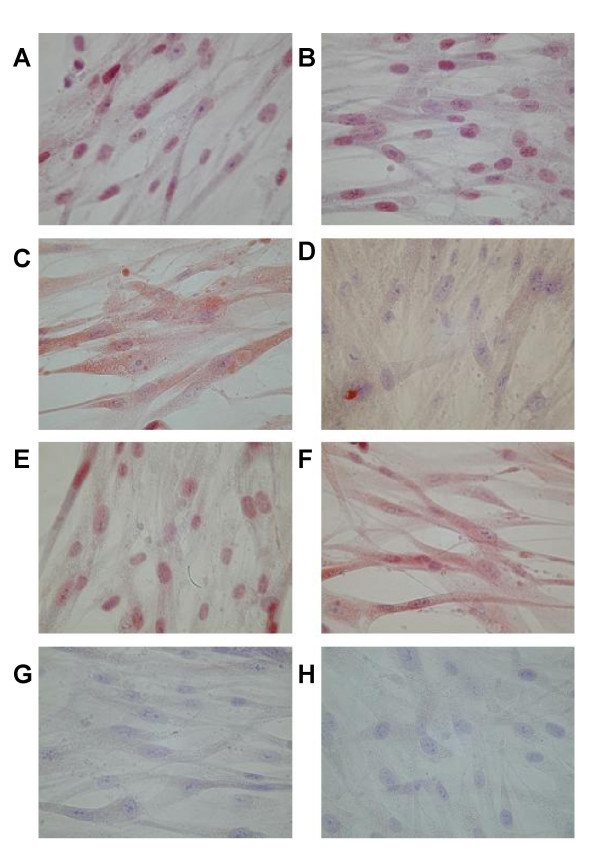
**Immunocytochemistry for 5-LO pathway enzymes and LT receptors in human bronchial fibroblasts**. Representative photomicrographs of fibroblasts from one donor. Fibroblasts were grown to confluence on coverslips and immunostained using primary antibodies against (**A**) 5-LO, (**B**) FLAP, (**C**) LTA_4 _hydrolase, (**D**) LTC_4 _synthase, (**E**) BLT1 receptors and (**F**) CysLT_1 _receptors. Staining with equivalent concentrations of (**G**) rabbit serum and (**H**) goat serum are shown as negative controls for A-E and F, respectively. Magnification x400. Positive immunostaining appears red (AEC) against the blue nuclear counterstain (Meyer's haematoxylin).

**Figure 4:**
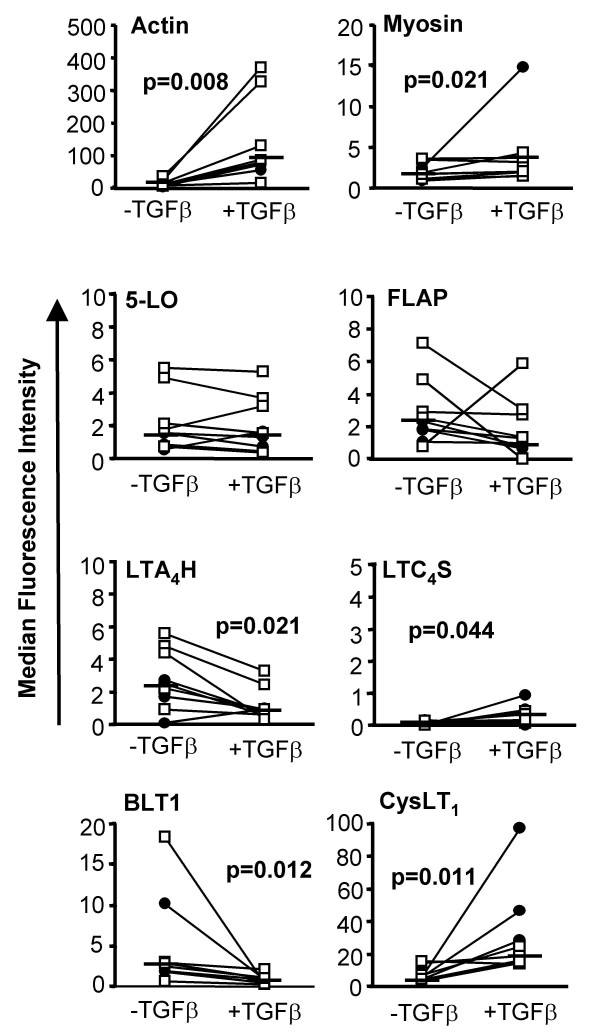
**Effects of TGF-β on expression of contractile proteins, 5-LO pathway enzymes and leukotriene receptors in fibroblasts from normal and asthmatic subjects**. Fibroblasts were cultured in the presence (+TGFβ) and absence (-TGFβ) of TGF-β_1 _(10 ng/ml) for 72 hours before staining with specific antibodies for actin, myosin, 5-LO, FLAP, LTA_4_H, LTC_4_S, BLT1 and CysLT_1_R and analysis by flow cytometry. Horizontal bars represent median values of all experiments where data points from normal donors are presented as a hollow square (n = 5) and asthmatic donors as a filled circle (n = 4).

### Isolation of peripheral blood leukocytes

In some experiments, peripheral blood leukocytes (PBL), obtained from normal venous blood by dextran sedimentation, were used as positive controls. Blood (20 ml) anti-coagulated with EDTA was mixed with 5% dextran (MW 70 kD) in PBS with 2% FCS. After sedimentation for 60 min, the leukocyte-rich upper layer underwent hypotonic red cell lysis in distilled water for 45s. Following centrifugation, PBL comprised 60–65% neutrophils, 30–40% mononuclear cells, 1–3% eosinophils and <1% basophils, as determined by Diff-Quik (Brownes Ltd, UK) staining, with a viability of >95% by Trypan Blue exclusion.

### Reverse-transcription PCR

Total RNA in cells grown in 10 cm^2 ^Petri dishes was isolated using the Trizol method (Gibco). Cells were homogenized in Trizol with glycogen and extracted with chloroform. After centrifugation, RNA in the upper aqueous phase was precipitated with isopropanol overnight at -20°C, washed twice in 80% ethanol and resuspended in DEPC-treated water. For first-strand cDNA synthesis, 2 μg RNA was incubated for one hour at 37°C in a total volume of 20 μl of DEPC-treated water with 2 μl of 10 × RT buffer, 2 μl of dNTP mix, 2 μl of oligo-dT primer (10 μM), 1 μl of RNase inhibitor (10 U/μl) and 1 μl of Omniscript reverse transcriptase, according to the Qiagen kit protocol.

PCR primer sequences were designed with Oligo Primer Analysis Software version 5 (National Biosciences, Inc) and synthesized by MWG Biotech (Milton Keynes, UK) (Table 1). PCR was performed with 2 μl of reverse transcription products in a total volume of 25 μl of DEPC-treated water, containing 10 × PCR buffer (2.5 μl), Mg^2+ ^(minimum 25 mM), dNTP (10 mM), Jumpstart (2.5 U/μl) and 1 μl of each upper and lower primer per tube (or 1.5 μl of GAPDH primers). Using a Peltier Thermal Cycler (PTC-225, MJ Research), thirty PCR cycles were carried out at 96°C for 5 min, 94°C for 30 s, 54°C (for CysLT_1_R), 58°C (for 5-LO, FLAP, and LTA_4 _hydrolase), 57°C (for GAPDH) or 64°C (for LTC_4_synthase) for 30 s, followed by 72°C for 7 min. Reaction products were separated on a 2% agarose gel and bands were visualized using ethidium bromide (0.1%). The gel was scanned using a Fluorimager 595 (Molecular Dynamics, Little Chalfont, UK) with Phoretix advanced software (Nonlinear Dynamics Ltd, Newcastle upon Tyne, UK). GAPDH was the positive control, and as a negative control, PCR was carried out in the absence of cDNA for each set of primers. Control experiments using genomic DNA instead of cDNA showed no bands for either the 5-LO, FLAP, LTA_4_H or LTC_4_S primers.

**Table 1:** Primer sequences for RT-PCR analysis of 5-LO pathway enzymes and receptors in human bronchial fibroblasts.

**Enzyme/Receptor**	**Primer Sequence**
**5-LO**	*upper *5'-TAC ATC GAG TTC CCC TGC TAC-3'
	*lower *5'-GTT CTT TAC GTC GGT GTT GCT-3'
**FLAP**	*upper *5'-GAT GCG TAC CCC ACT TTC CTC-3'
	*lower *5'-GAA TAT GCC AGC AAC GGA CAT-3'
**LTA**_4_**H**	*upper *5'-GCC CGA GAT AGT GGA TAC CTG-3'
	*lower *5'-TTT TTG CTC AAA GCG ATA GGA-3'
**LTC**_4_**S**	*upper *5'-CTG TGC GGC CTG GTC TAC CGT 3'
	*lower *5'-GGG AGG AAG TGG GCG AGC AG-3'
**CysLT**_1_	*upper *5'-CCG CTG CCT TTT TAG TCA GTT TC-3'
	*lower *5'-GGG TAC ATA AGT CAC GCT GGA CA-3'
**GAPDH**	*upper *5'-GCT TGT CAT CAA TGG AAA TCC-3'
	*lower *5'-AGG GGA TGC TGT TCT GGA GAG-3'

### Flow cytometry

For immunofluorescent staining of intracellular proteins (5-LO, FLAP, LTA_4 _hydrolase, LTC_4 _synthase, actin, myosin and vimentin) and the C-terminal intracellular epitope of the BLT1 receptor and the CysLT1 receptor, cells were detached from plastic ware with trypsin/EDTA and fixed in 4% paraformaldehyde in PBS for 30 min on ice. After washing in PBS, cells were treated with glycine buffer (0.1 M in PBS) for 10 min to prevent antibody/paraformaldehyde interactions. After two further washes in PBS, cells were permeabilised in PBS containing saponin (0.5%) and BSA (0.5%) for 30 min. Subsequent steps were performed in saponin-PBS to prevent reversal of permeabilisation. For CysLT_1 _(whole protein), immunofluorescent staining was performed on unfixed cells without permeabilisation. In this case cells were detached with non-enzymatic cell dissociation medium to prevent proteolysis of extracellular receptor epitopes and washed in FACS buffer (PBS containing 1% BSA and 0.1% sodium azide). All subsequent steps were also performed using FACS buffer.

Optimal dilutions of antibodies were prepared in the relevant buffer (with or without saponin) containing 10% human serum. Anti-human 5-LO, FLAP, LTA_4 _hydrolase, LTC_4 _synthase, and BLT1 antibodies were raised in rabbits, CysLT_1 _in goat, and actin, myosin and vimentin antibodies in mice. Cells were washed three times and incubated with optimal dilutions of the appropriate fluorescence-labeled secondary antibody (goat anti-rabbit/PE, rabbit anti-goat/FITC or rabbit anti-mouse/FITC) for 30 min. After washing, cells were re-suspended in 250 μl buffer and immunofluorescence was assessed by FACScan flow cytometer; analysis was by Cellquest software (Becton Dickinson).

### Immunoblotting

Immunoblotting for leukotriene pathway enzymes was performed essentially as described [18]. Cells were suspended in 250 μl protease inhibitor cocktail and sonicated on ice for three 10 s pulses at 16 μm (Soniprep). Total cellular protein concentrations were equalised to 1 mg/ml after analysis by DC protein assay (Bio-Rad, Hemel Hempstead, UK). SDS reducing buffer (0.06 M Tris, 10% SDS, 10% β-mercaptoethanol, 50% glycerol, 0.05% bromophenol blue) was added to the cell lysate (1:3) before heating at 80°C for 5 min. Electrophoresis of 5-LO and LTA_4 _hydrolase required 10% polyacrylamide gels, while FLAP and LTC_4_synthase were run on 12% gels. Each lane was loaded with 25 μl of cell lysate (or 5 μl molecular weight markers) and electrophoresed at 0.04 Amps for 45 min on Bio-Rad mini-Protean II equipment. Proteins were semi-dry transferred to nitrocellulose membrane (20 V, 20 min) and treated with PBS containing 5% dry milk powder overnight to reduce non-specific binding. For Western blotting, antibodies were made up in PBS with 0.05% Tween non-ionic detergent and 1% BSA. Optimally diluted primary antibodies were applied for 90 min before the membrane was washed (3 × 5 min) in PBS with 0.05% Tween. The secondary antibody (biotinylated swine-anti-rabbit IgG) was applied for 90 min. After washing, SAB-HRP conjugate was applied for 1 hour. After washing, protein bands were visualised using SuperPlus chemiluminescent substrate (Amersham Pharmacia Biotech, UK).

### Immunocytochemistry

Fibroblasts were cultured on glass coverslips for immunocytochemistry. When confluent, cells were washed in PBS, fixed in cold methanol for 10 min then washed again. To irreversibly block endogenous peroxidases, coverslips were incubated with 0.3% hydrogen peroxide solution with 0.1% sodium azide for 30 min at room temperature. After washing three times in TBS for 5 min, non-specific binding was reduced by incubation with DMEM containing 10% FCS and 1% BSA for 30 min. Rabbit or goat polyclonal primary antibodies were applied for 1 hour at titrated dilutions. After washing, biotinylated secondary antibody was applied for 1 hour followed by streptavidin-biotin-horseradish peroxidase conjugate for 1 hour. Immunostaining was visualized using AEC chromagen and the nuclei counterstained by Meyer's haematoxylin.

### Enzyme immunoassays

Cell culture supernatants were stored at -20°C before assay of LT concentrations using Biotrak LTC_4_/D_4_/E_4 _and LTB_4 _EIA kits in accordance with the manufacturer's instructions. The cysteinyl-LT EIA was calibrated with standard LTC_4 _from 0.75–48 pg/well, and used a polyclonal antiserum that cross-reacts with LTC_4 _(100%), LTD_4 _(100%), LTE_4 _(70%) and their 11-trans isomers, but negligibly with LTB_4 _(0.3%), various prostanoids or glutathione (<0.006%). The LTB_4 _EIA was calibrated with standard LTB_4 _from 0.30–40 pg/well and used a polyclonal antiserum that cross-reacts with LTB_4 _(100%), 20-OH-LTB_4 _(2%) and 6-trans-LTB_4 _(25.5%), but negligibly with LTC_4_, LTD_4_, or products of 12-LO or 15-LO. Following incubations, cells were detached and counted in order to express EIA results as pg/10^6 ^cells.

### Statistical analyses

Results are expressed as mean ± SEM, but Normality was not assumed and statistical comparisons were performed using non-parametric tests on SPSS version 12.0 for Windows (Microsoft Systems Inc). Paired comparisons were performed by Wilcoxon sign rank tests, and where the number of possible comparisons was large, overall significance was first assessed by a Friedman test. The Mann-Whitney *U *test was used to compare unpaired data between the asthmatic and normal subject groups. *p *≤ 0.05 was accepted as significant.

## Results

### Basal expression of LT pathway enzymes in human bronchial fibroblasts

To examine whether HBFs constitutively express leukotriene pathway enzymes, baseline levels of 5-LO, FLAP, LTA_4 _hydrolase and LTC_4 _synthase mRNA and protein were assessed by RT-PCR, SDS-PAGE/Western immunoblotting, immunocytochemistry and flow cytometry. A non-quantitative RT-PCR approach, using GAPDH as a positive control, was sufficient to establish the presence of mRNA transcripts for 5-LO, FLAP, LTA_4 _hydrolase and LTC_4 _synthase, as well as for the BLT1 and CysLT_1 _receptors in cultured fibroblasts (Figure 1A). The lengths of the amplicons generated were consistent with the expected values (124 bp, 210 bp, 291 bp, 155 bp and 248 bp respectively), and the LTC_4_S amplicons had the expected sequence.

SDS-PAGE/Western immunoblotting (Figure 1B) revealed that fibroblasts also constitutively expressed 5-LO, FLAP, LTA_4 _hydrolase and LTC_4 _synthase proteins, with immunopositive bands corresponding to the expected molecular weights of approximately 80 kDa, 18 kDa, 70 kDa and 18 kDa respectively. 5-LO and FLAP in fibroblasts co-migrated with recombinant 5-LO and FLAP standards, and the intensities of immunostaining for all four enzymes were similar to those observed in equal amounts of total cellular protein extracted from PBL (Figure 1B).

Fibroblasts from both subject groups expressed similar levels of mRNA and protein for the 5-LO pathway enzymes and leukotriene receptors (normal subjects, n = 5; mild-moderate asthmatic patients, n = 5).

Baseline expression of 5-LO pathway enzymes and receptors in fibroblasts was also assessed by flow cytometry. Flow cytometry plots for the biosynthetic proteins (5-LO, FLAP, LTA_4 _hydrolase, LTC_4 _synthase) and receptors (BLT1, CysLT_1_) are shown in Figure 2; these are representative plots of control data from the nine subjects shown in Table 3. The presence of CysLT_1 _was further confirmed by flow cytometry with a polyclonal antibody recognizing the C-terminal amino acids 318–337 (Cayman Chemical), in the presence and absence of a CysLT_1 _receptor blocking peptide (Cayman Chemical) corresponding to the target sequence of this antibody. There were no inherent differences in surface expression of 5-LO pathway enzymes and leukotriene receptors in fibroblasts from mild-moderate asthmatic patients (n = 5) compared with normal subjects (n = 5)(Table 2).

**Table 2:** Immunofluorescence for 5-LO pathway enzymes and LT receptors in fibroblasts from normal and asthmatic donors.

**Enzyme/Receptor**	Normal*(Mean MFI ± SEM n = 5)	**Asthmatic (Mean MFI ± SEM n = 5)**
**5-LO**	9.7 ± 2.8	8.5 ± 3.1
**FLAP**	12.3 ± 3.8	10.5 ± 4.5
**LTA**_4_**H**	7.8 ± 1.9	8.6 ± 3.7
**LTC**_4_**S**	0.97 ± 0.4	2.1 ± 1.4
**BLT1**	10.5 ± 3.1	6.7 ± 3.6
**CysLT**_1_	7.9 ± 1.4	5.5 ± 2.4

* Fibroblasts from 5 normal subjects and 5 asthmatic patients were stained with specific antibodies to 5-LO, FLAP, LTA_4_H, LTC_4_S, BLT1 and CysLT_1_R before analysis by flow cytometry.

**Table 3:** Effects of cytokines, autacoids and leukotrienes on 5-LO pathway enzymes and LT receptors in HBFs.

	Control*	**EGF**	**Cytomix**	**LTB**_4_	**LTD**_4_	**BK**	**HIS**
**5-LO**	9.1 ± 2.0	8.8 ± 2.4	9.4 ± 3.6	10.5 ± 3.2	9.5 ± 2.4	10.8 ± 2.9	6.4 ± 1.9^†^
**FLAP**	11.4 ± 2.8	11.3 ± 4.2	12.2 ± 3.7	10.5 ± 2.2	11.8 ± 3.2	11.2 ± 2.7	11.3 ± 3.6
**LTA**_4_**H**	8.2 ± 2.0	9.3 ± 2.3	10.1 ± 2.4	9.2 ± 2.2	9.1 ± 1.9	5.8 ± 1.1	9.8 ± 2.5
**LTC**_4_**S**	1.5 ± 0.7	1.2 ± 0.3	0.8 ± 0.5	0.8 ± 0.3^‡^	1.0 ± 0.5	1.0 ± 0.3	1.1 ± 0.4
**BLT1**	8.8 ± 2.3	14.2 ± 5.0	13.5 ± 4.9	12.8 ± 4.6	10.9 ± 4.5	11.5 ± 4.2	11.2 ± 4.7
**CysLT**_1_	6.7 ± 1.4	7.5 ± 2.1	14.3 ± 4.5	12.0 ± 5.7	7.8 ± 1.5	8.5 ± 1.4	9.9 ± 1.7

* Fibroblasts were cultured for 24 hours with either EGF (10 ng/ml), a combination of IL-1β, TNF-α and IFN-γ (Cytomix, each at 10 ng/ml), LTB_4 _(10 nM), LTD_4 _(10 nM), bradykinin (BK, 10 μM), histamine (HIS, 10 μM) or control medium before staining with specific antibodies and analysis by flow cytometry.

^† ^p = 0.047 versus Control

^‡ ^p = 0.036 versus Control

Immunocytochemistry was used to visualize cellular expression of the 5-LO pathway enzymes and LT receptors; representative photomicrographs of normal fibroblasts are shown in Figure 3. Positive AEC immunostaining (red) was observed for 5-LO, FLAP, LTA_4 _hydrolase, LTC_4 _synthase, BLT1 receptors and CysLT_1 _receptors (Figure 3A–F), while antibody-negative controls showed no immunostaining (Figure 3G, H).

### Effect of transforming growth factor-β on expression of 5-LO pathway enzymes and LT receptors in human bronchial fibroblasts

Stimulation with transforming growth factor-β_1_(TGF-β_1_) is known to transform fibroblasts into a more contractile, myofibroblast-like phenotype [19], accompanied by increased expression of the contractile proteins α-smooth muscle actin and myosin. The effect of TGF-β_1 _(10 ng/ml, 72 hours) on the expression of 5-LO pathway enzymes and LT receptors was therefore examined in fibroblasts by flow cytometry. As expected, TGF-β_1 _stimulation increased the median immunofluorescence intensity (MFI) for actin nearly 10-fold from 14.6 ± 3.4 to 135.1 ± 41.8 units (p = 0.008) and doubled that for myosin from 2.0 ± 0.3 to 3.9 ± 1.4 (p = 0.021)(n = 9). Treatment with TGF-β_1 _did not alter the MFI for 5-LO (2.2 ± 0.6) or FLAP (2.8 ± 0.7), but LTC_4 _synthase immunofluorescence increased five-fold from 0.06 ± 0.02 to 0.3 ± 0.1 units (p = 0.044), while CysLT_1_R increased four-fold from 7.3 ± 1.5 to 30.6 ± 9.0 (p = 0.011). Conversely, expression of LTA_4 _hydrolase and BLT1 was significantly reduced by TGF-β_1 _(MFI from 2.8 ± 0.6 to 1.2 ± 0.3 units, p = 0.021, and from 5.1 ± 2.2 to 0.9 ± 0.2, p = 0.012, respectively) (Figure 4). No significant differences were found between the mild-moderate asthmatic and normal subject groups in their responses to TGF-β_1 _(Figure 4).

### Effect of TGF-β_1 _on A23187-induced synthesis of cysteinyl-leukotrienes and LTB_4_

**Figure 5:**
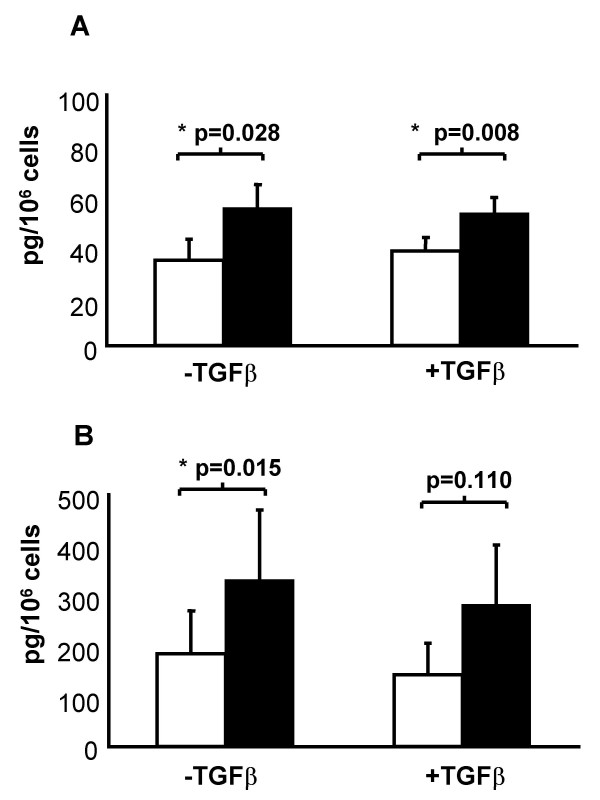
**Synthesis of cysteinyl-LTs and LTB_4 _by human bronchial fibroblasts**. Fibroblasts were cultured for 72 hours in the presence or absence of TGF-β_1 _(10 ng/ml) before they were washed and stimulated for a further 2 hours with calcium ionophore A23187 (1 μM, filled column) or vehicle (open column). Levels of total cysteinyl-LTs (LTC_4_D_4_E_4_) (**A**) and LTB_4 _(**B**) in culture supernatants were then measured by EIA. Mean ± SEM (n = 9).

To investigate the enzymatic activity of the 5-LO pathway in human bronchial fibroblasts, production of LTB_4 _and total cysteinyl-LTs was quantified by EIAs. Following 72 hours incubation with or without TGF-β_1 _(10 ng/ml), fibroblasts were incubated for a further two hours in the presence or absence of the divalent cation ionophore A23187 (1 μM). Immunoreactivity for cysteinyl-LTs and LTB_4 _was readily detectable in fibroblast supernatants from unstimulated cells (33.8 ± 8.3 and 182.1 ± 86.9 pg/10^6 ^cells respectively). Stimulation with ionophore significantly increased the mean production of cysteinyl-LTs by approximately 60% to 54.2 ± 9.4 pg/10^6 ^cells (p = 0.028), and production of LTB_4 _increased by 80% to 327 ± 150 pg/10^6 ^cells (p = 0.015; n = 9) (Figure 5). Culture for 72 hours with TGF-β_1 _had no effect on the amounts of LTB_4 _and LTC_4 _released either in unstimulated cells or in those stimulated with A23187 (Figure 5). Fibroblasts from both subject groups produced similar amounts of cysteinyl-LTs and of LTB_4 _and responded to stimulation to a similar degree.

### Effects of growth factors, inflammatory cytokines and autacoids on expression of 5-LO pathway enzymes and LT receptors

Fibroblasts were cultured for 24 hours with either epidermal growth factor (EGF) (10 ng/ml), a combination of IL-1β, TNF-α and IFN-γ (each at 10 ng/ml), LTB_4 _(10 nM), LTD_4 _(10 nM), bradykinin (10 μM) or histamine (10 μM) and flow cytometry was used to quantify immunofluorescence for the leukotriene pathway enzymes and receptors. Compared to control medium, none of these stimuli significantly increased the expression of 5-LO, FLAP, LTA_4 _hydrolase, LTC_4 _synthase, BLT1 or CysLT_1 _(Table 3). Treatment with histamine for 24 hours caused a small but significant decrease in 5-LO (MFI from 9.1 ± 2.0 to 6.4 ± 1.9, p = 0.036) and treatment with LTB_4 _caused a significant decrease in LTC_4_S (MFI from 1.5 ± 0.7 to 0.8 ± 0.3, p = 0.047); these changes may have arisen from the large number of statistical comparisons performed on Table 3 as Friedman's test for non-parametric multiple comparisons suggested no overall effect. No significant differences were found between the subject groups in their responses to treatment with ionophore, growth factors, inflammatory cytokines or autacoids.

### Effects of dexamethasone and MK-571 on expression of 5-LO pathway enzymes and LT receptors

Flow cytometry was used to quantify the effects of culturing fibroblasts for 24 hours with the glucocorticoid dexamethasone (1 μM) or the CysLT_1 _receptor antagonist MK-571 (10 nM). Friedman tests indicated no overall effects of either drug on any 5-LO pathway protein or LT receptor, although individual comparisons using Wilcoxon sign rank tests suggested that MK-571 modestly reduced the expression of FLAP (p < 0.05) and increased expression of LTC_4 _synthase (p < 0.043), while dexamethasone produced non-significant reductions of 25–53% in immunofluorescence for 5-LO (p < 0.07) and LTA_4 _hydrolase (p < 0.07) (n = 9)(Table 4). No significant differences were found between the subject groups in their responses to dexamethasone or MK-571.

**Table 4:** Effects of dexamethasone and MK-571 on 5-LO pathway enzymes and LT receptors in HBFs.

**Enzymes/Receptors**	Control*	**Dexamethasone**	**MK-571**
**5-LO**	7.6 ± 2.2	5.7 ± 2.0^†^	7.5 ± 2.6
**FLAP**	9.3 ± 3.3	6.3 ± 2.5	5.3 ± 2.0^‡^
**LTA**_4_**H**	7.8 ± 2.1	5.3 ± 1.6^†^	5.2 ± 0.9
**LTC**_4_**S**	0.3 ± 0.2	0.7 ± 0.4	0.5 ± 0.2^‡^
**BLT1**	4.8 ± 1.1	4.5 ± 1.3	3.9 ± 0.9
**CysLT**_1_	11.7 ± 3.5	5.6 ± 1.4	16.0 ± 6.9

* Fibroblasts were cultured for 24 hours with the glucocorticoid dexamethasone (1 μM), the CysLT_1 _receptor antagonist MK-571 (10 nM), or control medium before staining with specific antibodies then analyzed by flow cytometry (Mean ± SEM, n = 9).

^† ^Non-significant trend towards reduced expression compared to control, p = 0.066.

^‡ ^p ≤ 0.05 *versus *Control.

## Discussion

Cysteinyl-leukotrienes are key mediators of bronchoconstriction and airway eosinophilia in the asthmatic lung. Studies in animal models have also implicated these autacoids in airway fibrosis [10,11], and exogenous cysteinyl-LTs can enhance proliferation and collagen release by fibroblasts *in vitro *[8,9]. Our study investigated the hypothesis that human bronchial fibroblasts may express the 5-LO pathway and directly generate cysteinyl-LTs and the chemotactic agent LTB_4_.

Approaches at the levels of mRNA, protein and lipid product demonstrated constitutive expression and activity of the key LT biosynthetic enzymes 5-LO, FLAP, LTA_4 _hydrolase and LTC_4 _synthase in fibroblasts from normal and asthmatic bronchi. The data thus confirm that primary HBF express the distal enzyme (LTA_4 _hydrolase) required to complete LTB_4 _synthesis [20], but also show for the first time that HBF also express the proximal enzymes 5-LO and FLAP required to initiate synthesis of the unstable intermediate LTA_4_, as well as the terminal LTC_4 _synthase required to generate the first of the cysteinyl-LTs. Constitutive expression of each enzyme was revealed at the mRNA level by RT-PCR (Figure 1A), and also at the level of immunoreactive protein by Western blotting (Figure 1B), flow cytometry (Figure 2), and immunocytochemistry (Figure 3). The primary antibodies used in these latter experiments are highly selective; the rabbit antibody to 5-LO does not cross-react with other human lipoxygenases, such as 12-LO or 15-LO [14]. The rabbit antiserum to FLAP does not cross-react with LTC_4 _synthase, despite the strong sequence homology of the two enzymes [21], and conversely, the LTC_4 _synthase antiserum does not cross-react with FLAP nor with microsomal glutathione-S-transferase type II [16].

The enzymatic activity of the 5-LO pathway was confirmed using highly-specific enzyme immunoassays for released cysteinyl-LTs and LTB_4_. Each sub-family of LT was readily detected in fibroblast-con ditioned media from normal and asthmatic donors, both spontaneously and in increased amounts after stimulation with the calcium ionophore A23187 (Figure 5), which activates phospholipase A_2 _and 5-LO by increasing intracellular Ca^2+^. Although the levels of expression of 5-LO, FLAP, LTA_4 _hydrolase and LTC_4 _synthase proteins in HBF were comparable to those detected in peripheral blood leukocytes (Figure 1B), the generation of their lipid products after ionophore stimulation was modest in comparison [22,23]. This finding suggests that a factor other than the amounts of 5-LO and its downstream enzymes may determine the maximal amounts of LT produced, including the activity of the proximal phospholipases A_2 _that mobilize arachidonate from membrane phospholipids. Further experiments in the presence of exogenous arachidonate may elucidate this question.

The data nevertheless add cysteinyl-LTs and LTB_4 _to the growing list of autacoids, cytokines and growth factors known to be generated by primary HBF, including prostaglandin E_2_, IL-1, IL-6, IL-13, granulocyte-colony stimulating factor, granulocyte macrophage-colony stimulating factor and eotaxin [1,24]. As the predominant stromal cell-type in the bronchial submucosa, even low levels of production of cysteinyl-LTs by HBF in asthma may contribute substantially to bronchial smooth muscle contraction, microvascular permeability, mucus hypersecretion and eosinophil recruitment [5]. The latter may arise through direct effects at CysLT_1 _receptors on the eosinophil surface, or indirectly via the secondary release of eotaxin from fibroblasts triggered by autocrine or paracrine cysteinyl-LTs [24]. Similarly, the generation of LTB_4 _by HBF may contribute to infiltration of the airway by neutrophils and monocytes in response to inhaled irritants [25,26].

Our study showed for the first time that primary fibroblasts from normal and asthmatic bronchi constitutively express both mRNA and protein for CysLT_1 _receptors under baseline conditions (Figures 1,2,3, Table 2). Human nasal polyp fibroblasts and the human fetal lung fibroblast cell line HFL-1 are not thought to constitutively express CysLT_1 _receptor protein[24,27], although its expression on HFL-1 cells is inducible by IL-13 [24]. CysLT_1 _receptor expression may be absent in fetal lung fibroblasts *in vivo *or it may be lost during the 27 passages endured by the HFL-1 cell line. The HBF in our study were examined between passages five and seven. The CysLT_1 _receptors observed on adult HBF may mediate the effects of autocrine or exogenous cysteinyl-LTs on fibroblast proliferation and collagen deposition [8,9], and also mediate fibroblast apoptosis induced by oxidant injury [28]. As well as CysLT_1 _receptors, HBF also constitutively expressed the BLT1 receptor, which is normally thought to be restricted to myeloid leukocytes in which it mediates chemotaxis to LTB_4 _[13]. This may underlie the ability of nanomolar concentrations of LTB_4 _to induce chemotaxis in human fibroblasts *in vitro *[29,30].

Having established the baseline expression of 5-LO pathway enzymes and LT receptors in HBFs, we then investigated whether their expression is modulated when fibroblasts are transformed to myofibroblasts, a phenotype intermediate between fibroblasts and myocytes characterized by contractility and extracellular matrix deposition. The most potent inducer of myofibroblast differentiation is TGF-β [17,31]. Compared to cultures in DMEM/10%FCS, which itself contains some serum-derived TGF-β, treatment for three days with exogenous TGF-β_1 _(10 ng/ml) up-regulated expression of the contractile proteins, α-smooth muscle actin and myosin heavy chain, it was shown that this is associated with marked increases in LTC_4 _synthase and CysLT_1 _receptors, and with a decline in expression of LTA_4 _hydrolase and BLT1 receptors (Figure 4). This suggests that the myofibroblast phenotype may be linked with an enhanced capacity to synthesize and respond to cysteinyl-LTs, and with a corresponding decline in ability to synthesize and respond to LTB_4_. That this was not associated with changes at the level of synthesized lipid product under the conditions used suggests an additional level of regulation, perhaps at the level of the proximal phospholipases, that requires further investigation. As the activities of LTB_4 _and cysteinyl-LTs may be regarded as chemotactic and pro-fibrotic respectively, these changes are nevertheless consistent with the association of the fibroblast with inflammatory leukocyte recruitment and activation and the myofibroblast with contractility.

We next investigated the *in vitro *effects of a range of factors associated with asthma and fibrosis that might modulate the LT pathway in fibroblasts (Table 3). At the concentrations used and in 24-hour incubations, there were no meaningful effects of leukotrienes B_4 _and D_4_, the CysLT_1 _antagonist MK-571, histamine, bradykinin, EGF, or pro-inflammatory cytokines (IL-1β, TNF-α and IFN-γ) on the expression of 5-LO pathway proteins and LT receptors. In the light of the ability of IL-13 to up-regulate CysLT_1 _in HFL-1 cells [24], and the contrasting failure of IL-4 to induce CysLT_1 _or the 5-LO/LTC_4 _synthase pathway in nasal polyp fibroblasts [27], it would be of interest to investigate the effects of these Th2-type cytokines on 5-LO pathway enzymes and LT receptors in primary lung fibroblasts. The impact of glucocorticoids on the 5-LO pathway is variable, with paradoxical upregulation of 5-LO and FLAP being seen in leukocytes [18,32]. The lack of effect of dexamethasone on 5-LO pathway enzymes in HBF (Table 4) is consistent with their lack of effect on leukocyte production of leukotrienes and the failure of glucocorticoid therapy to alter LT levels *in vivo *[33].

Lung fibroblasts from asthmatic subjects have been reported to display persistent phenotypic anomalies compared to normal fibroblasts, including exaggerated proliferative responses even in the absence of mitogens [34]. We hypothesized that asthmatic fibroblasts might also have an upregulated 5-LO pathway or enhanced LT receptor expression. An enhanced capacity for LT synthesis has been observed in the mast cells and PBL of asthmatics compared with normal cells, associated with increased expression of FLAP and other 5-LO pathway enzymes[18,23,35]. However, in HBF, there were no differences between the normal and asthmatic donors in basal immunofluorescence for 5-LO pathway enzymes or LT receptors (Table 2), or in their capacity to synthesize LTB_4 _and cysteinyl-LTs, nor did any differences emerge when cells from the two groups were treated with TGF-β_1 _or with calcium ionophore A23187. This may reflect the relatively small numbers of subjects in our study (n = 5 in each group), or such differences might exist *in vivo *but be lost during cell culture even within 5–7 passages.

## Conclusion

Our study has revealed that in contrast to human fetal lung fibroblast cell lines [24], primary fibroblasts from the bronchi of normal and asthmatic adults constitutively express CysLT_1 _receptors, and that this receptor is up-regulated by transformation of fibroblasts to a contractile myofibroblast phenotype, while the constitutive expression of BLT1 receptors may explain fibroblast chemotactic responses to LTB_4_. The study has identified for the first time in human bronchial fibroblasts the presence of a complete 5-LO enzymatic pathway that actively synthesizes cysteinyl-LTs and LTB_4 _at baseline, and in increased amounts after calcium-dependent activation. In the airways, in disorders such as asthma, these LTs might promote migration and collagen deposition by fibroblasts in an autocrine manner leading to sub-epithelial fibrosis, as well as promoting other remodeling responses in adjacent tissues including the proliferation of epithelial cells and airway smooth muscle [7].

## Competing interests

The author(s) declare that they have no competing interests.

## Authors' contributions

AJJ carried out all laboratory-based experiments, participated in the design of the study and in the analysis and data interpretation, and drafted the manuscript. APS participated in the conception and design of the study, the analysis and interpretation of the data and critical evaluation of the manuscript. AMC participated in the analysis and interpretation of the data and critical evaluation of the manuscript. STH participated in the design of the study and critical evaluation of the manuscript. JFP participated in the critical evaluation of the manuscript. All authors read and approved the final manuscript.
